# Correction to “Suspected large gall bladder mucocele extending from right hypochondrium up to right iliac region turned out to be gall bladder perforation in a patient with schizophrenia: A rare case report and literature review”

**DOI:** 10.1002/ccr3.9424

**Published:** 2024-09-05

Kumar A, Upadhyay P, Kumar H, Mukherjee D, Tiwari D, Akilimali A. Suspected large gall bladder mucocele extending from right hypochondrium up to right iliac region turned out to be gall bladder perforation in a patient with schizophrenia: A rare case report and literature review. Clin Case Rep. 2024;12:e9284. doi:10.1002/ccr3.9284

In authors' contact details, email id of author Divyansh Tiwari, that is, “divyanshtiwari99@gmail.com” was incorrect. This should have read as “divyanshtiwarii99@gmail.com.”

We apologize for this error.

